# Characterization of the microvascular cerebral blood flow response to obstructive apneic events during night sleep

**DOI:** 10.1117/1.NPh.5.4.045003

**Published:** 2018-11-03

**Authors:** Peyman Zirak, Clara Gregori-Pla, Igor Blanco, Ana Fortuna, Gianluca Cotta, Pau Bramon, Isabel Serra, Anna Mola, Jordi Solà-Soler, Beatriz F. Giraldo-Giraldo, Turgut Durduran, Mercedes Mayos

**Affiliations:** a ICFO-Institut de Ciències Fotòniques, Barcelona Institute of Science and Technology, Barcelona, Spain; b Hospital de la Santa Creu i Sant Pau, Department of Respiratory Medicine, Sleep Unit, Barcelona, Spain; c Centre de Recerca Matemàtica (CRM), Bellaterra, Spain; d Universitat Politècnica de Catalunya (UPC)-Barcelona Tech, Department of Automatic Control (ESAII), Barcelona, Spain; e The Barcelona Institute of Science and Technology, Institute for Bioengineering of Catalonia (IBEC), Barcelona, Spain; f Centro de Investigación Biomédica en Red de Bioingeniería, Biomateriales y Nanomedicina (CIBER-BBN), Zaragoza, Spain; g Institució Catalana de Recerca i Estudis Avançats (ICREA), Barcelona, Spain; h CIBER Enfermedades Respiratorias (CibeRes) (CB06/06), Madrid, Spain

**Keywords:** sleep disorder breathing, cerebral blood flow, brain perfusion, diffuse correlation spectroscopy

## Abstract

Obstructive apnea causes periodic changes in cerebral and systemic hemodynamics, which may contribute to the increased risk of cerebrovascular disease of patients with obstructive sleep apnea (OSA) syndrome. The improved understanding of the consequences of an apneic event on the brain perfusion may improve our knowledge of these consequences and then allow for the development of preventive strategies. Our aim was to characterize the typical microvascular, cortical cerebral blood flow (CBF) changes in an OSA population during an apneic event. Sixteen patients (age 58±8  years, 75% male) with a high risk of severe OSA were measured with a polysomnography device and with diffuse correlation spectroscopy (DCS) during one night of sleep with 1365 obstructive apneic events detected. All patients were later confirmed to suffer from severe OSA syndrome with a mean of 83±15 apneas and hypopneas per hour. DCS has been shown to be able to characterize the microvascular CBF response to each event with a sufficient contrast-to-noise ratio to reveal its dynamics. It has also revealed that an apnea causes a peak increase of microvascular CBF (30±17%) at the end of the event followed by a drop (−20±12%) similar to what was observed in macrovascular CBF velocity of the middle cerebral artery. This study paves the way for the utilization of DCS for further studies on these populations.

## 1 Introduction

Obstructive sleep apnea (OSA) is characterized by the intermittent and repetitive collapse of the upper airway during sleep with simultaneous respiratory effort. Symptoms such as headache, sleepiness, fatigue, depression and difficulties in keeping concentration are frequent in patients with OSA.^1^ Even more, OSA has been related to an increased risk of cardiovascular and cerebrovascular diseases, such as systemic hypertension, atrial fibrillation, and cerebral stroke,^2^^–^^5^ as well as to increased mortality.^6^^,^^7^ The key factors involved are the repetitive intermittent hypoxia, the increased sympathetic activity, the sleep fragmentation, and the periodic cerebral hemodynamic changes but further understanding is desirable.^8^^,^^9^

Previously, the apnea-induced changes of cerebral hemodynamics have been studied and characterized by several groups through the measurement of the cerebral blood flow velocity (CBFV) in the middle cerebral artery by transcranial Doppler (TCD).^10^^–^^14^ In Bålfors and Franklin^10^ study, both CBFV and the mean arterial blood pressure showed a biphasic pattern, where during the apnea a gradual increase of both CBFV and mean arterial blood pressure was observed followed by a sudden drop after the end of the apneic event. These hemodynamic changes are rapid and, therefore, many modalities for CBF measurement are not applicable, limiting the literature to studies, where the macrovascular CBFV was measured by TCD. Few studies have also used microvascular cerebral blood oxygenation measured by near-infrared diffuse optical spectroscopy (NIRS-DOS) as a surrogate.^15^^–^^18^

However, neither TCD nor NIRS-DOS can measure the actual microvascular cerebral blood flow (CBF) in the brain, which is a desirable parameter since it provides direct information about the health of the brain,^19^ acts as biomarker of cerebral autoregulation,^20^ and is a key parameter to measure the oxygen metabolism.^21^^–^^23^ This is what led us to adopt an emerging technology, diffuse correlation spectroscopy (DCS), to measure local, microvascular CBF on the brain cortex noninvasively at the bed-side.^24^^,^^25^ DCS utilizes near-infrared light like NIRS-DOS but relies on the speckle statistics of the laser light to characterize red blood cell motion. To the best of our knowledge, only one study attempted to measure night sleep changes by DCS in OSA patients^26^ but could not characterize individual apneic events, presumably due to technical limitations.

In this study, we have used a DCS device to evaluate and characterize the individual apnea-induced hemodynamic changes of CBF measured continuously in patients with severe OSA simultaneously by using DCS and polysomnography (PSG).

## 2 Methods

This study was conducted at a referral Sleep Unit (Department of Respiratory Medicine, Hospital de la Santa Creu i Sant Pau) in Barcelona, Spain. The study protocol was approved by the local ethical committee (EC/11/001/1166). All participants gave their informed written consent. It was part of a larger study involving other modalities.

Patients were referred to a sleep study at the unit because of being at a high risk of severe OSA according to the Epworth sleeping scale^27^ results, other clinical symptoms, and the results of a previous home-use nocturnal pulse oximetry session.^28^

Those who were older than 80 years, had previous or current continuous positive air pressure (CPAP) treatment,^29^ had chronic obstructive pulmonary or neuromuscular diseases, a previous ischemic stroke, or who refused to participate in the study were excluded. Demographic and clinical characteristics were obtained for all participants. A pre-established questionnaire was used to collect demographic variables including their medical history, cardiovascular risk factors, and current medications. Diagnosis of arterial hypertension was defined as having ≥140  mmHg systolic blood pressure and/or ≥90  mmHg diastolic blood pressure.^30^

All patients were asked to arrive at the Sleep Unit at 19:00 on the study date. They were instructed to avoid caffeinated or alcoholic beverages 24 h previously to the measurement. PSG monitors and optical probes were placed as explained below. Concurrent optical and PSG data were acquired during the night sleep.

If the obstructive apnea or hypopnea index (AHI; number of apneas and hypopneas per hour of sleep) was greater than 30 after about 4 h of sleep, the clinical technician fixed a CPAP mouth-nose mask to find the correct air pressure for preventing apneas (called split-night PSG). For those patients with split-night PSG, only the data recording of the first 4 h of night sleep without CPAP were then used for further analysis since there are practically no apneas during CPAP use.

### 2.1 Overnight Polysomnography

PSG (Siesta Compumedics, Melbourne, Australia) sensors were wirelessly connected to the monitoring room. Among other variables, PSG included the recording of the oronasal flow (by a thermistor and a nasal cannula), the thoracic and abdominal movements (by a respiratory inductance plethysmography band), the heart rate (HR; by electrography chest leads and calculated from the electrocardiogram as described in Ref. 31), and the arterial oxygen saturation (${\mathrm{SpO}}_{2}$; by pulse oximetry).

PSG data were postprocessed and manually scored by the sleep technicians according to the Spanish Sleep group recommendations,^29^ which, among other things, describe the rules for scoring respiratory events. Sleep technicians determined the start and end time points of each apneic event, identified the apnea types (i.e., obstructive apnea, hypopnea, mixed apnea, and central apnea) and calculated, among other parameters, the percentage of total sleep time with ${\mathrm{SpO}}_{2}$ lower than 90% (CT90), the 4% oxygen desaturation index (ODI4), and the AHI. From these variables, the diagnosis of OSA and the high degree of severity of these patients were confirmed or rejected after our recruitment. Due to the different pathophysiology of each type of apneic event, and for simplicity, only obstructive apneas were used for the analysis of this study.

### 2.2 Determination of the Cerebral Blood Flow by Diffuse Correlation Spectroscopy

Microvascular CBF during the whole night sleep was continuously assessed by a custom-built DCS system that was previously described.^25^^,^^32^^,^^33^ Briefly, the DCS consisted of a mode-hope free, long-coherence-length, continuous-wave laser at 785 nm and eight single photon avalanche photodiode detectors, whose outputs were fed to a custom-built hardware autocorrelator. DCS uses the intensity autocorrelation of the diffuse light to evaluate the motion of the scatterers, i.e., the red blood cells.^24^ The intensity autocorrelation data are then fitted by a physical model of the photon diffusion in tissues to determine a blood flow index (BFI), which is recorded as a continuous variable. The BFI (${\mathrm{cm}}^{2}/s$) is not a measure of absolute blood flow in traditional units. Even though under controlled situations, the absolute values are proportional to the absolute blood flow,^34^ the relative changes are more reliable and have been shown to be quantitative.^25^^,^^35^ Therefore, we report relative changes in this work.

The averaging time of the DCS measurement in each patient was adjusted from 1 to 3 s during the first minutes of the measurement in order to maximize the signal-to-noise ratio for the rest of the sleep measurement. In order to coregister the DCS data with the PSG variables, a transistor–transistor logic signal was generated through a digital output channel, which was fed into the PSG device and was used as a marker to synchronize DCS and all PSG variables.

The optical probe was made of custom built, 90-deg bent fibers of 2 mm of external diameter and consisted of a source fiber of a core of 200  μm and a detector fiber bundle of four single-mode fibers of a core of 5.6  μm. The source–detector separation was 2.5 cm. We have assumed that the hemodynamic changes in the brain are homogeneous bilaterally and, for patient comfort, we opted to fix a single DCS probe on the right forehead of the patient. The probe was placed over the patient’s forehead, properly fixed to avoid the movement of the patient, and allowed the placement and removal of the CPAP mask when necessary with the minimum impact possible on the optical measurement. A black elastic band was attached to the standard CPAP head frame to fix the optical probe and the CPAP mask on the head.

### 2.3 Group and Individual Analysis of Apnea Induced Cerebral Blood Flow, Heart Rate, and Arterial Oxygen Saturation Changes

Individual apneic events were characterized by the percent relative CBF change (ΔrCBF), defined as $\Delta r\mathrm{CBF}=(\frac{\mathrm{BFI}}{{\mathrm{BFI}}_{\mathrm{bl}}}-1)\times 100$, where ${\mathrm{BFI}}_{\mathrm{bl}}$ is the average of the cerebral BFI from 30 s before the apnea start up to 30 s after the apnea end. This choice for ${\mathrm{BFI}}_{\mathrm{bl}}$ was used to account for the possible changes in the absolute CBF at different stages of sleep and to correct for slight changes in the probe position during the whole night of sleep. We note that we have taken a similar approach to systemic variables too, i.e., ΔrHR was defined as $\Delta r\mathrm{HR}=(\frac{\mathrm{HR}}{{\mathrm{HR}}_{\mathrm{bl}}}-1)\times 100$, and $\Delta {\mathrm{SpO}}_{2}$ was defined as $\Delta {\mathrm{SpO}}_{2}={\mathrm{SpO}}_{2}-{\mathrm{SpO}}_{2\mathrm{bl}}$.

In order to discard the CBF, HR, and ${\mathrm{SpO}}_{2}$ responses to obstructive apneic events with a low signal quality or with movement artifacts during the measurement, all responses were studied by previously developed methods for outlier detection.^36^^,^^37^ These allowed us to find the responses that exhibited a different time behavior or that presented higher or lower magnitude values than the majority. Also, the outlier detection method^36^^,^^37^ allowed us to reduce the effect of outliers that exist not only due to measurement issues but also because of uncontrolled physiological outliers (e.g., mixture of two events, other physiological alterations). Each variable (CBF, HR, or ${\mathrm{SpO}}_{2}$) was analyzed independently. For instance, if the ΔrCBF response for one apnea was classified as an outlier, it did not imply that ΔrHR and/or $\Delta {\mathrm{SpO}}_{2}$ response for the same apnea were also classified as outliers. We do not expect this to cause any errors in the data analysis due to the large number of events that were analyzed. The outlier detection procedure was implemented in Ref. 38 using the “fda.usc”^37^ package and the R function “Outliergram.”^36^

**Fig. 1:**
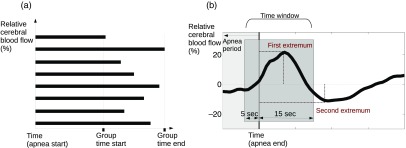
(a) Visualization of the averaging of the different apneas grouped by their duration. These apneas from the same duration range will be averaged for obtaining a canonical apnea shape. (b) Characterization of individual apneas, where the apnea end is considered as a pivot point. The light gray region indicates the apneic event. The dark gray region indicates the time window used to find the first extremum value. The first and second extrema are labeled.

After removing the outliers from our database, all the remaining apneic events for each variable (ΔrCBF, ΔrHR, or $\Delta {\mathrm{SpO}}_{2}$) of all patients were averaged in order to visualize representative cerebral and systemic dynamics of obstructive apneas. The averaging was performed by (1) selecting the start and the end of each apnea based on PSG measurements using the established criteria (see above), (2) calculating the ΔrCBF, ΔrHR, or $\Delta {\mathrm{SpO}}_{2}$ traces for each apnea, as explained previously, (3) aligning the data considering as the pivot point the start of each individual apnea, as shown in Fig. 1(a), and (4) grouping and averaging all apneas within a given range of apnea duration. Four groups were used based on their duration; apneas shorter than or equal to 15 s, apneas longer than 15 and up-to 30 s, apneas longer than 30 and up-to 45 s, and apneas longer than 45 and up-to 60 s. There were apneas of varying lengths in each group and, if an apnea was shorter than the full duration, it did not contribute to the remaining average. This grouping before averaging was done since the apnea lengths vary from 10 s up to around a minute and, even though it is not perfect, grouping by duration allowed us to see more details of the dynamics.

This heterogeneity of the duration of apneas did not allow us to analyze the full duration of the single apnea induced ΔrCBF, ΔrHR, or $\Delta {\mathrm{SpO}}_{2}$ changes. Instead, we have considered the apnea end as a pivot point to calculate each parameter. The parameters associated to each obstructive apneic event were considered as a function dependent on time [ΔrCBF (time), ΔrHR (time), and $\Delta {\mathrm{SpO}}_{2}$ (time)], and then, the relative extrema of these functions along a specific time interval relative to the apnea end were calculated. The positive extrema are referenced as “peak” values, and the negative as “drop” values. The time windows to find these extrema were from −5 to 15 s for the first extremum on ΔrCBF [see Fig. 1(b) as an example], from 0 to 15 s for the ΔrHR, and from 5 to 35 s for the $\Delta {\mathrm{SpO}}_{2}$. In order to visualize the possible link between the hypoxemia present in these patients and the CBF, also the second extremum that was outside this window was considered for ΔrCBF. These time windows were selected from the literature^10^^,^^39^ and also by visual observation of all the apneas plotted together from −30  s to 60 s in order to include the majority of the peak/drop values. This analysis was performed with MATLAB 2012a (Mathworks, Massachusetts).

The association between the calculated ΔrCBF, ΔrHR, and $\Delta {\mathrm{SpO}}_{2}$ extrema to the apnea duration (from the PSG) was analyzed by adjusting a linear mixed-effect model.^40^ The patient identifier was used as a random factor, the parameter apnea duration was the fixed effect, and the positive and negative extrema (previously defined as “peaks” and “drops”) of the apnea time response on variables ΔrCBF, ΔrHR, and $\Delta {\mathrm{SpO}}_{2}$ were the predictors. The linear mixed-effect analysis was carried out in the R programming language and environment^40^ using the “nlme” package. The associations between the mean of the previously calculated ΔrCBF, ΔrHR, and $\Delta {\mathrm{SpO}}_{2}$ extrema responses for each patient with gender, age, and body mass index (BMI) (one by one) were analyzed by performing simple linear models. The demographic parameters were the fixed effects and the mean calculated extrema were the predictors. The residuals of the models were checked for linearity by plotting the standard residuals versus the predicted means. Residuals were inspected for deviations from homoscedasticity. Also, residuals were inspected for deviations from normality by means of histograms and also by means of Q–Q plots. The presence of influential data points was also inspected.

The Wilcoxon signed-rank test was used to check if ΔrCBF, ΔrHR, and $\Delta {\mathrm{SpO}}_{2}$ peaks and drops for each grouping of apneas by duration were different from zero.

A p-value<0.05 was considered to be statistically significant.

## 3 Results

We have included 16 patients with high risk of severe OSA. Fourteen patients were studied with a split-night PSG and two patients with overnight PSG. All sixteen patients were diagnosed with severe OSA according to the criteria described above.

The microvascular CBF during the whole night of sleep was continuously assessed by DCS with a range of 0.9 to 3.1 (1.5±0.5, mean±standard deviation) second time-resolution in order to maximize the signal-to-noise ratio. The time-resolution was decided during a baseline test, as mentioned in Sec. 2. The typical count rate for these patients was from 50 to 150 kHz.

A total of 3817 apneic events were identified including 1365 (36%) obstructive apneic events. The DCS recording in two patients was discarded (14% of total obstructive apneic events) due to synchronization failure between the PSG and the DCS. A part of the HR of different patients was discarded due to low electrocardiogram data quality recording (15% of total obstructive apneic events). The ${\mathrm{SpO}}_{2}$ recording in one patient was discarded (9% of total obstructive apneic events) due to the detachment of the pulse oximeter during the main part of the recording. After removing the outliers, 87% obstructive apneic events were considered for the CBF, 90% events for the HR, and 88% events for the ${\mathrm{SpO}}_{2}$. Further clarification of the total of number of apneas considered for the analysis is given in Sec. 5.

Table 1 shows the demographic, clinical, and polysomnographic characteristics of the subjects. The table shows that this is a relatively homogeneous group of patients with a very severe OSA syndrome, commonly associated with a high percentage of cardiovascular and metabolic comorbidity. The severity of OSA syndrome in our cohort is shown by an AHI higher than 30, and high values of CT90 and ODI4. The prevalence of hypertension in our sample was 62.3%, which is consistent with the results of other studies.^41^ Four patients received beta blockers, which may cause alterations in the HR.^42^ There was no other relevant use of medications.

**Table 1:** Demographic, clinical, and polysomnographic characteristics of the patient population. Values are reported in median (interquartile range) or frequency (%).

	OSA patients (n=16)
Age (years)	57 (52–64.5)
Males n (%)	12 (75)
BMI (${\mathrm{kg}/\mathrm{cm}}^{2}$)	34 (32–37.5)
Epworth	9.5 (7.5–15.5)
AHT n (%)	10 (62.5)
Smokers n (%)	13 (81)
Diabetes n (%)	5 (31.25)
Dyslipidemia n (%)	3 (18.75)
AHI (n./h)	85 (76–94)
Mean ${\mathrm{SpO}}_{2}$ (%)	92 (90.5–93.5)
CT90 (%)	23 (12–33)
ODI4 (%)	74 (65–85)
Total number of apneas detected by PSG n	3817
Obstructive apneas n (%)	1365 (36)
Hypopneas n (%)	1918 (50)
Mixed apneas n (%)	358 (9)
Central apneas n (%)	176 (5)

Note: OSA, obstructive sleep apnea; BMI, body mass index; AHT, arterial hypertension; AHI, apnea-hypopnea index; ${\mathrm{SpO}}_{2}$, arterial oxygen saturation by pulse oximetry; CT90, % of time ${\mathrm{SpO}}_{2}$ lower than 90% of total sleep time; ODI4, 4% oxygen desaturation index.

**Fig. 2:**
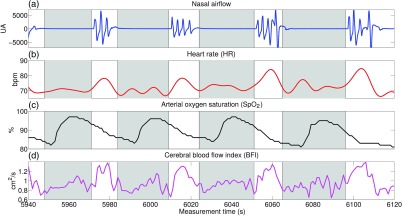
(a) Nasal airflow, (b) heart rate, (c) arterial oxygen saturation, and (d) cerebral blood flow index dynamics during 3 min of night sleep for one representative subject. The gray regions between two vertical lines indicate the obstructive apneic events.

As an example of the apnea effect on systemic variables and CBF, Fig. 2 shows 3 min of continuous BFI measurement together with nasal airflow, HR, and ${\mathrm{SpO}}_{2}$ changes for one representative patient. BFI has been plotted here instead of ΔrCBF, since this is calculated from a specific baseline of each individual apnea, where the baseline corresponds to a preapnea period of 30 s from the start up-to a postapnea period up-to 30 s from the end. From the PSG recording, we can see that ${\mathrm{SpO}}_{2}$ shows a drop with a delay relative to the apneic event. In this time period with frequent apneas, characteristic of patients with severe OSA, the ${\mathrm{SpO}}_{2}$ drop of the previous apnea is in the apnea period of the next event. It can also be observed that the HR starts to rise when the breathing restarts after a period of cessation. BFI also shows a similar behavior as the HR.

**Fig. 3:**
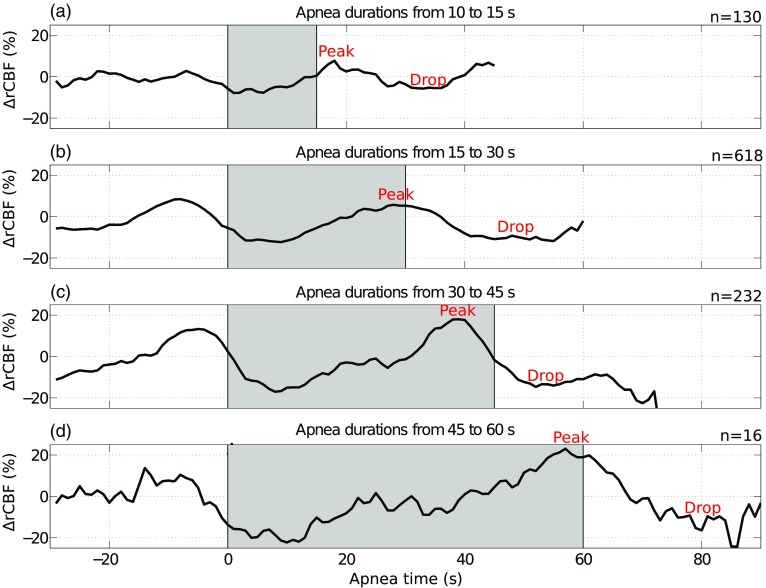
Mean relative cerebral blood flow changes (ΔrCBF) during obstructive apneic events for apnea durations of: (a) 10 to 15 s; (b) 15 to 30 s; (c) 30 to 45 s; and (d) 45 to 60 s. The gray regions between two vertical lines indicate the start of the events up-to the end of the longest events in each group. The total number of averaged apneas for each subfigure is included at the top right. The peaks and drops representative for the mean apnea hemodynamics response to obstructive apneic events for each group are labeled. See Fig. 1(a) for the visualization of the different apneas grouped by their duration before averaging.

To better understand the general response of CBF to obstructive apneic events, ΔrCBF measurements during obstructive apneas were grouped depending on their duration (as explained in Sec. 2) and averaged, as shown in Fig. 3. It can be observed that the mean of the different apnea-duration groups follows a similar pattern: a ΔrCBF increase toward the apnea end followed by a drop (in ΔrCBF).

**Fig. 4:**
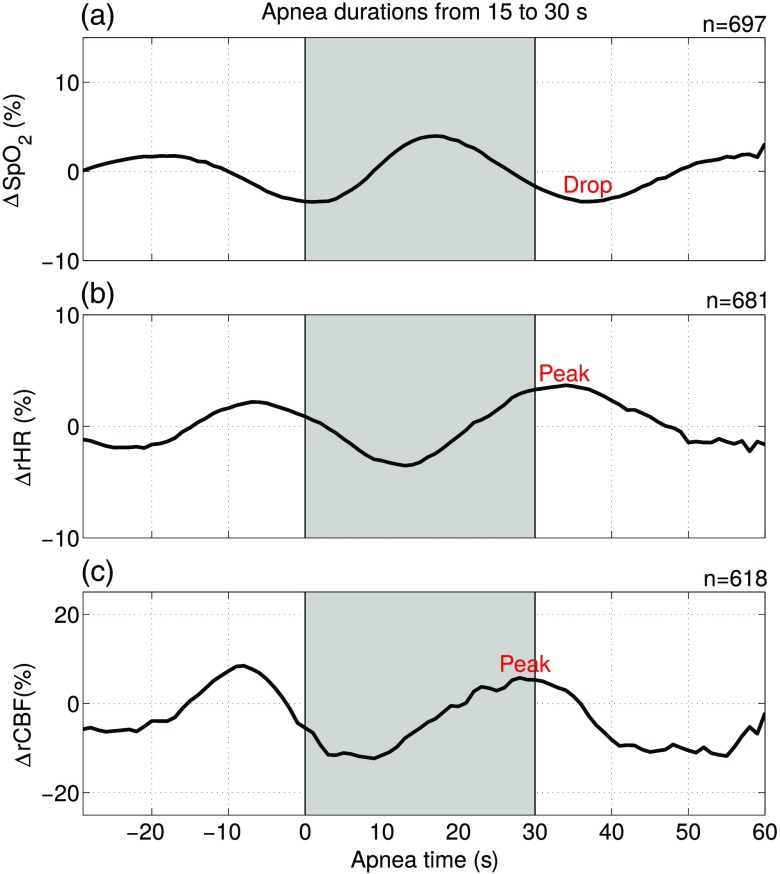
(a) Mean change of arterial oxygen saturation ($\Delta {\mathrm{SpO}}_{2}$), (b) of relative heart rate (ΔrHR), and (c) of relative cerebral blood flow (ΔrCBF), for apnea durations from 15 up-to 30 s. The gray regions between two vertical lines indicate the start of the events up-to the end of the longest events of 30 s. The total number of averaged apneas for each subfigure is included at the top right. The peaks and drops representative for the mean $\Delta {\mathrm{SpO}}_{2}$, ΔrHR, and ΔrCBF response to obstructive apneic events are labeled. See Fig. 1(a) for the visualization of the different apneas grouped by their duration before averaging.

Apnea duration range from 15 up-to 30 s has been chosen for further visualization of the data since it has the highest number of events. The peak observed in CBF in Fig. 3 is also observed in the HR in Fig. 4, whereas the ${\mathrm{SpO}}_{2}$ shows a drop. We can also observe in Fig. 2 that cerebral and systemic variables are not constant during preapnea periods. This effect is clearly evident in the peaks/drops right before or at the start of the apnea in Figs. 3 and 4. This is attributed to the presence of a previous apneic event equal or less than 30 s prior to the start of the evaluated event, which was the case for 80% (n=3054) of all the events detected by PSG, i.e., the subject’s physiology did not yet stabilize. According to this, and following the literature, in order to characterize the response to a given apnea, we have considered only the CBF peaks/drops, HR peaks, and ${\mathrm{SpO}}_{2}$ drops near the end of the apnea or in the postapnea period (as explained in Sec. 2).

**Fig. 5:**
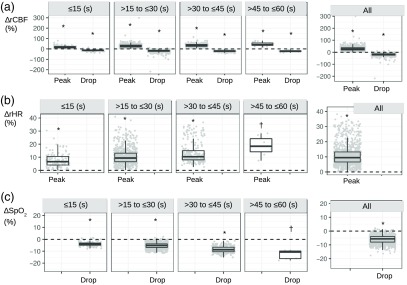
(a) Relative cerebral blood flow changes (ΔrCBF), (b) relative heart rate changes (ΔrHR), and (c) arterial oxygen saturation changes ($\Delta {\mathrm{SpO}}_{2}$) peaks and/or drops are shown, divided in different apnea group durations and for all apneas. These are also summarized in Table 2. (*) indicates that the group is statistically different from zero with p<0.001. (†) indicates that the group is statistically different from zero with p<0.05.

Figure 5 and Table 2 show the individual data points and average amounts of peaks/drops for cerebral and systemic variables (ΔrCBF, ΔrHR, and $\Delta {\mathrm{SpO}}_{2}$) grouped by apnea duration. All ΔrCBF, ΔrHR, and $\Delta {\mathrm{SpO}}_{2}$ peaks and drops for each grouping of apneas by duration were statistically different from zero. Microvascular CBF increased by a mean of 30±17% at the end of the event followed by a drop of −20±12%. HR, as expected, increased by −11±7%. Also, ${\mathrm{SpO}}_{2}$, as expected, decreased by −13±4%.

**Table 2:** Mean±standard deviation values for the amount of the peak or drop close to the apnea end for the different apneas grouped by their duration and for all apneas.

Apnea duration (s)	ΔrCBF			ΔrHR		$\Delta {\mathrm{SpO}}_{2}$	
	n (%)	Peak (%)	Drop (%)	n (%)	Peak (%)	n (%)	Drop (%)
≤15	130 (13)	22±15	−19±16	116 (11)	8±5	126 (11)	−4±2
>15 to ≤30	618 (62)	29±18	−20±13	681 (65)	11±6	697 (64)	−6±4
>30 to ≤45	238 (24)	35±15	−21±7	236 (23)	13±8	258 (24)	−9±6
>45 to ≤60	16 (1)	45±20	−22±9	7 (1)	19±8	7 (1)	−13±4
All, 24±8	1002 (100)	30±17	−20±12	1040 (100)	11±7	1088 (100)	−6±4

Note: ΔrCBF, relative cerebral blood flow change; ΔrHR, relative heart rate change; $\Delta {\mathrm{SpO}}_{2}$, arterial oxygen saturation change by pulse oximetry.

When fitting a linear model with the ΔrCBF peak or the ΔrHR peak as the dependent parameter and the apnea duration as the predictor parameter, positive statistically significant associations (β=0.5 and β=0.4, respectively) were found (p<0.001) for both dependent parameters. When the dependent parameter was the ΔrCBF drop or the $\Delta {\mathrm{SpO}}_{2}$ drop, negative statistically significant associations (β=−0.2 and β=−0.2, respectively) were found (p<0.001) for both dependent parameters. Females, in comparison to males, showed a larger CBF response (β=9.9, p=0.040). Older age was associated to smaller a ${\mathrm{SpO}}_{2}$ response (β=−0.2, p=0.004). No statistically significant associations were found with the BMI (p>0.05).

## 4 Discussion

In this work, we have demonstrated the successful assessment of microvascular CBF during individual obstructive apneic events by noninvasive, continuous DCS measurements. All subjects tolerated the study during the whole-night sleep showing the suitability of the technique for bed-side continuous CBF monitoring over long time periods and its compatibility with standard PSG monitoring.

Our first finding was that DCS results had the sufficient contrast-to-noise ratio in order to enable us to measure the dynamics of microvascular CBF during obstructive apneic events in a synchronized manner with systemic variables, as illustrated in Fig. 2. HR and ${\mathrm{SpO}}_{2}$ followed the expected dynamics according to the literature.^15^^,^^16^^,^^39^^,^^43^ CBF showed a similar behavior as HR. There is only one study that has also measured microvascular CBF in OSA patients continuously with DCS^26^ during night sleep. However, apnea cerebral hemodynamics were not characterized, instead, only 2-min time periods with apneas and 2-min time periods with no apneas were compared in order to see altered variability of the microvascular hemodynamics with or without apneas.

Our second finding revealed a steep rise and a peak of microvascular ΔrCBF toward or after the end of an apnea, followed by a drop. Figure 4 indicates that the ΔrCBF and ΔrHR traces are similar and are in-phase. This could suggest that we are primarily measuring the extracerebral contributions instead of the cerebral contribution, since, in principle, cerebral signals are not directly driven by heart-rate changes, i.e., the cerebral signals are autoregulated. However, the literature supports this type of correlation between HR changes and the cerebral signals during an apnea. For example, the reported microvascular CBF changes measured by DCS follow the same patterns of those of middle cerebral artery CBFV measured by TCD^10^^,^^13^^,^^14^ showing a peak close to the end of the apnea. In addition to the similarity of their temporal profile, these ΔrCBF and CBFV peaks are in agreement within variability of the both methods. The 14.6±14% peak change in CBFV right after the apnea end by Bålfors and Franklin^10^ is similar to our microvascular ΔrCBF values of 30±17% for obstructive apnea, as can be seen in Table 2. Also, Alex et al.^13^ found similar peaks in CBFV of 22% to 42%. However, other authors have reported larger CBFV peaks. Klingelhöfer et al.^11^ found changes of CBFV of 19% to 219% and Siebler and Nachtmann^14^ found a mean CBFV peak during apnea of 142% compared with the baseline CBFV. The differences of these last studies with our results may be related to the longer apnea durations and to different normalization of the data. The ΔrCBF drops after the apnea end are also in agreement with the CBFV drops found in the bibliography.^10^^,^^11^ These results tell us that there is a decrease in cerebral perfusion due to an apneic event. If these intermittent decreases lead to ischemia, they can cause hypoxic/ischemic brain injury, especially if cerebrovascular reactivity and regulation are impaired.^44^

Another point about the extracerebral contamination is that DCS in adult brain with this source–detector separation has been validated against other measures of CBF in different studies, where it was demonstrated that the relative changes in different challenges follow the intracerebral signals closely.^25^^,^^45^^–^^47^

However, despite these arguments, we cannot rule out the possibility that microvascular and macrovascular changes diverge and that extracerebral signals have strongly impressed themselves on the DCS signals. Future studies are needed to study this point.

We have observed (Figs. 2 and 3) that cerebral hemodynamics in the pre- and during-apnea periods are not stable as it has been previously observed due to the influence of the previous apnea.^15^^,^^16^^,^^39^^,^^43^ 80% (n=3054) of the total events (i.e., obstructive apneas, mixed apneas, hypopneas and central apneas) are followed by the next episode within 30 s or less, hence, we expect that the effects of the previous events overlap with the next apnea. This is because the rapid succession of events does not allow ample time for the physiology to recover as observed by Bålfors and Franklin,^10^ where they have reported that it took up to 60 s for CBFV to return to baseline after the apnea termination.

**Fig. 6:**
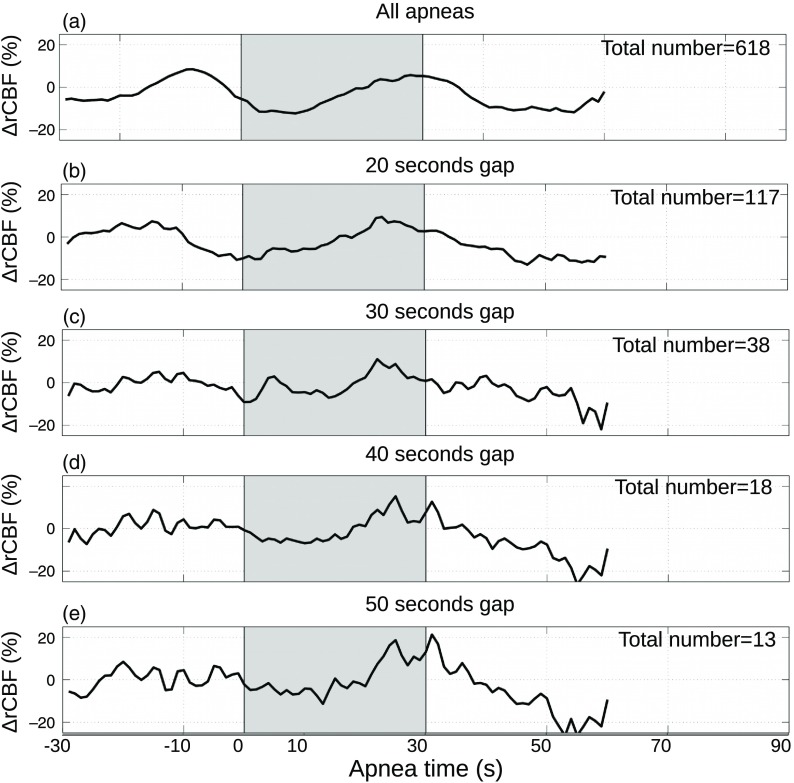
Mean relative cerebral blood flow changes (ΔrCBF) of apneas longer than 15 and up-to 30 s. (a) Average of all obstructive events, (b) those with no previous event 20 s before, (c) 30 s before, (d) 40 s before, and (e) 50 s before. The gray regions between two vertical lines indicates the start of the events up-to the end of the longest event of 30 s. The total number of averaged apneas for each panel is included at the top right.

We have attempted to resolve this by isolating apneas by forcing different lengths of minimum gaps between the events, however, in this group of patients with a severe condition, due to the high frequency of repetitive events, only a small group of apneas could be isolated, as shown in Fig. 6, and no final conclusions could be drawn about what would have happened if there had been no overlapping apneas.

The CBF peak and drop amplitudes that are characteristic of each apneic event were associated with the apnea duration. The association of the peak with the apnea duration has also been observed previously for CBFV in the middle cerebral artery.^13^ About the systemic variables, a correlation of desaturation depth with apnea duration has been observed previously by several authors.^48^^,^^49^ Also, the abrupt HR increase immediately after obstructive apnoeas has been documented,^50^ and recently, in a preliminary work, we have already observed a correlation between HR excursion and the duration of apneas.^31^ These results tell us that the longer the apnea duration, the bigger is its effect on systemic variables, but also, on the microvascular cerebral hemodynamics.

About the gender effect that was observed, where females showed larger CBF responses, this is in contrast to Edlow et al.,^51^ who has reported a smaller CBF response to HOB manipulation for females in the healthy population. It is difficult to know whether this is due to a smaller head circumference and a smaller scalp-to-brain-distance, hence a smaller extracerebral effect or not. We also note that we did not observe a BMI effect. However, Peppard et al.^52^ observed an association between BMI and ${\mathrm{SpO}}_{2}$ decreases. The BMI was quite homogeneous for our group (32 to 37.5), which may have hidden this relationship. About the age effect that was observed, older age has already been associated to smaller apnea ${\mathrm{SpO}}_{2}$ responses, as predicted in the literature.^53^

Finally, we have discarded several apneas (13% for CBF, 10% for HR, and 12% for ${\mathrm{SpO}}_{2}$; see Sec. 5) as outliers. Similar percentages of apneas were removed between CBF and the PSG variables (HR and ${\mathrm{SpO}}_{2}$) and, therefore, these support the idea that the DCS signal has the quality needed in the clinics.

Our study has some potential limitations that should be taken in consideration. First, the contribution from the extracerebral tissues could not be assessed independently since our probe lacked a short source–detector separation. A multidistance source–detector separation probe and pressure modulation algorithms^54^ should be considered in future studies. We do note that a source–detector separation of 2.5 cm has been found to be a good compromise and was validated in numerous studies.^25^^,^^47^^,^^46^ Second, the absorption and reduced scattering coefficients have been considered as constant along the study. While significant changes in the reduced scattering coefficient can affect the DCS results, they are not expected during an apnea. The changes in the absorption coefficient due to an apnea have a minimal effect on the DCS signal.^24^^,^^47^ Third, there are additional factors to consider to go deeper into the physiology of the relationship between the systemic physiology and microvascular CBF changes, such as the effects of different sleep states, arousals, leg movement, and others sleep events. The detailed analysis is beyond the scope of this paper and will be a point of future studies. Finally, our findings correspond to a group of patients with very severe OSA, which implies that these results are not necessarily extrapolated to the different OSA severities. However, at the same time, it strengthens the validity of our results for patients with severe OSA.

In summary, we have demonstrated that DCS is a suitable technology for bedside and continuous monitoring of the microvascular ΔrCBF during sleep. We were able to obtain sufficient signal-to-noise ratio to reveal the dynamics and the canonical shape of the microvascular CBF changes. We were then also able to characterize each CBF peak and the following drop in each obstructive sleep apneic event, as well as to visualize the apnea-induced cerebral and systemic hemodynamics simultaneously in patients with severe OSA. This work, to our best knowledge, is the first characterization of the microvascular CBF during an OSA.

## 5 Appendix

Not all the apneic events detected by the PSG technique have been used for the data plotting and analysis. Table 3 shows the different steps from the initial number of apneas detected by the PSG to the final number of apneas considered.

**Table 3:** Total number of apneas considered from the PSG detection and the apneas considered for the analysis for different steps. The total number of events remaining after each step and its percentage (%) are reported. Step 1, total events detected by PSG. Step 2, obstructive apneic events detected by PSG. Step 3, obstructive apneic events detected by PSG and recorded by each technique. Step 4, obstructive apneic events detected by PSG and recorded with each technique after removing the outliers.

Step		CBF	HR	${\mathrm{SpO}}_{2}$
1	Total apneas detected by PSG, n (%)	3817 (100)	3817 (100)	3817 (100)
2	Obstructive apneas detected by PSG, n (%) of step 1	1365 (36)	1365 (36)	1365 (36)
3	Obstructive apneas, n (%) of step 2	1150 (84)	1161 (85)	1239 (91)
4	Obstructive apneas after outlier removal, n (%) of step 3	1002 (87)	1040 (90)	1088 (88)

Note: CBF, cerebral blood flow; HR, heart rate; ${\mathrm{SpO}}_{2}$, arterial oxygen saturation by pulse oximetry; PSG, polysomonography.
